# Sex and suicide: The curious case of Toll-like receptors

**DOI:** 10.1371/journal.pbio.3000663

**Published:** 2020-03-23

**Authors:** Paulo A. Navarro-Costa, Antoine Molaro, Chandra S. Misra, Colin D. Meiklejohn, Peter J. Ellis

**Affiliations:** 1 Instituto Gulbenkian de Ciência, Oeiras, Portugal; 2 Instituto de Saúde Ambiental, Faculdade de Medicina, Universidade de Lisboa, Lisbon, Portugal; 3 Division of Basic Sciences, Fred Hutchinson Cancer Research Center, Seattle, Washington, United States of America; 4 Instituto de Tecnologia Química e Biológica, Oeiras, Portugal; 5 School of Biological Sciences, University of Nebraska-Lincoln, Lincoln, Nebraska, United States of America; 6 School of Biosciences, University of Kent, Canterbury, United Kingdom

## Abstract

During in vitro fertilisation (IVF), pharmacological activation of the murine X chromosome–encoded receptor proteins Toll-like receptor (TLR) 7 and TLR8 reportedly results in male-biased litters by selectively disrupting the motility of X-bearing sperm cells. Thus—in the context of agonist treatment during IVF—these receptors act as ‘suicidal’ segregation distorters that impair their own transmission to the next generation. Such behaviour would, from an evolutionary perspective, be strongly selected against if present during natural fertilisation. Consequently, TLR7/8 biology in vivo must differ significantly from this in vitro situation to allow these genes to persist in the genome. Here, we use our current understanding of male germ cell biology and TLR function as a starting point to explore the mechanistic and evolutionary aspects of this apparent paradox.

## Abstract 2

This Unsolved Mystery article considers the paradoxical case of TLR7 and TLR8, two apparently "suicidal" genes recently reported as controlling offspring sex ratio. How can it be possible for X-linked genes to selectively inhibit the success of X-bearing sperm, without immediately being eliminated by natural selection?

## Introduction

Umehara and colleagues recently reported that the membrane-associated receptor proteins Toll-like receptor (TLR) 7 and TLR8 can be used to separate mouse sperm cell fractions enriched either for X or Y chromosome–bearing sperm [1]. According to this study, X-encoded TLR7/8 are predominantly expressed in X-bearing sperm, and the activation of these receptors reduces sperm cell motility. More specifically, following treatment of sperm cells with known TLR7/8 agonists and collection of fractions enriched for slower- and faster-moving cells, the slower cell fraction yielded female-biased offspring after in vitro fertilisation (IVF), whereas the faster fraction was biased toward male offspring. Ensuing press attention has focused on the application of this technique for sex selection in livestock and perhaps even in humans, a possibility which, if realised, could lead to largely unpredictable societal consequences. Setting aside these wider implications, the observations of Umehara and colleagues also present an unsolved mystery in that TLR7/8 appear to function as ‘suicidal’ segregation distorters. A gene that impedes its own reproductive success should be rapidly selected against and unable to persist in the population [2]. Below, we address various aspects of this mystery, focusing specifically on two key questions: (1) How are TLR7/8 specifically enriched in X-bearing sperm cells? And (2) what are the possible evolutionary consequences of this peculiar haploid selection mechanism?

## Till spermiation do us part: Male germ cells develop as a syncytium

**Fig 1:**
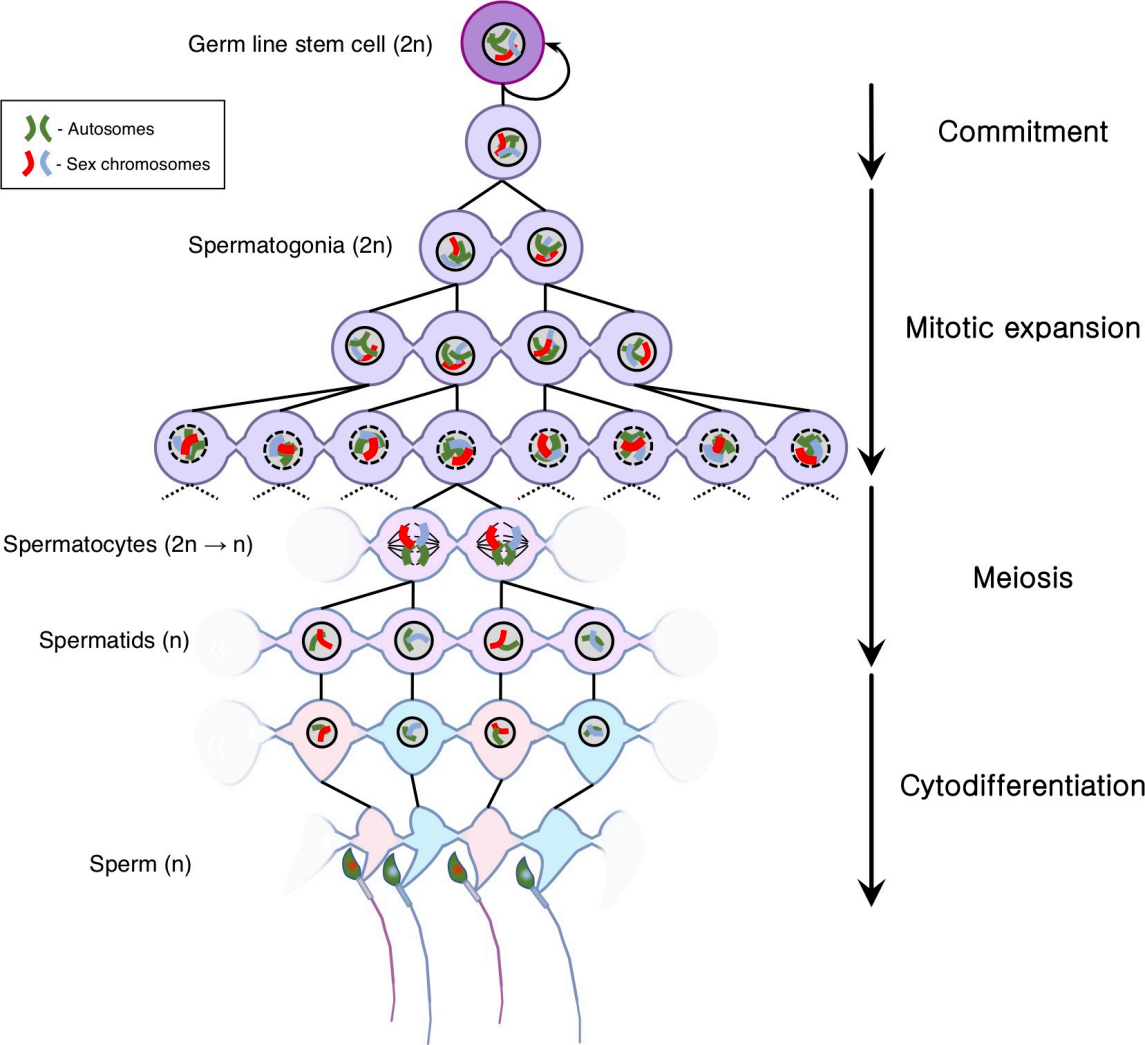
Intercellular bridges connect developing male germ cells. These bridges are established after commitment to differentiation and ensure cytoplasmic continuity between the progeny of all subsequent mitotic and meiotic divisions. Sperm cell individualisation only occurs immediately prior to the release of the fully differentiated sperm from the gametogenic tissue (spermiation). The intercellular bridges are large enough to accommodate the flow of mRNAs, proteins, and even organelles between cells. Note that the degree of sharing of any given product is determined by a source–sink process, potentially resulting in increased levels near the site of production (as illustrated by the two cytoplasm hues in postmeiotic cells, matching the sex chromosome they contain). mRNA, messenger RNA.

Spermatogenesis is a cell differentiation programme through which diploid sperm cell precursors become haploid, fertilisation-capable male gametes. Despite the often striking morphological differences between male gametes from different species [3], the fundamental stages of spermatogenesis are evolutionarily conserved and include (1) the commitment of male germ line stem cells to differentiation, (2) the amplification of committed cells through a series of mitotic divisions, (3) a reduction of ploidy (segregation of homologs) through meiosis, and (4) a final cytodifferentiation programme that converts postmeiotic cells into mature male gametes. Another evolutionarily conserved feature of spermatogenesis is that upon commitment to differentiation, all subsequent germ cell divisions (both mitotic and meiotic) are characterised by incomplete cytokinesis [4,5]. This results in the formation of intercellular bridges that provide cytoplasmic continuity between all daughter cells stemming from a single committed precursor (**Fig 1**). Such continuity is only lost right at the end of spermatogenesis, when fully differentiated male germ cells are released from the testicular tissue as individualised gametes (a process known as spermiation). Spermatogenesis is therefore a syncytial process, with developing sperm cells remaining interconnected in cysts of up to hundreds of cells, depending on the species [6].

## A bridge over uncharted waters

The functional significance of the intercellular bridges between male germ cells is still a matter of debate. One of the most generally accepted hypotheses is that the cytoplasmic continuity they provide equalises the genomic contents of postmeiotic cells [7], thus rendering postmeiotic cells phenotypically diploid [8]. Support for this hypothesis comes from the observation that, in postmeiotic cells, X-encoded transcripts pass through intercellular bridges in the form of protein–messenger RNA (mRNA) complexes [9] and that, in general, both the X and Y chromosomes encode postmeiotically expressed genes that are essential for sperm cytodifferentiation and function, e.g., [10–12]. Consequently, it is undeniable that postmeiotic equalisation of X- and Y-encoded gene products across the syncytium must be essential for fertility in mammals. Nevertheless, this cannot be the only or even the primary reason for the presence of these bridges, for three reasons: Firstly, cytoplasmic bridges are present in all species studied to date, including species without differentiated sex chromosomes [13], species with female rather than male heterogamety [14], and species with haploid males [15]. Secondly, as discussed above, bridges are generated during mitotic as well as meiotic divisions occurring in the germ line. Thirdly, disruption of the bridges leads to germ cell arrest and apoptosis before the completion of the first meiotic division—i.e., at a point when developing sperm cells are still genetically equivalent [16]. These observations prove that additional functional constraints must underlie the development of these peculiar communication channels. In this regard, an alternative and nonexclusive hypothesis is that the cytoplasmic bridges regulate developmental synchronisation between groups of germ cells, aiding the identification and elimination of cells with aberrant progression [17].

The intriguing nature of cytoplasmic bridges extends from their hypothetical functions to the very nature of the communication they mediate. Indeed, RNA trafficking within male germ cell syncytia is an important and understudied area of developmental biology. From a mechanistic perspective, transcript trafficking appears to be in part mediated by the chromatoid body, a non-membrane-bound organelle formed within each germ cell at the end of meiosis [18]. This large ribonucleoprotein structure has been shown by electron microscopy to shuttle between developing spermatids via the intercellular bridges [19]. The dynamic localisation of the chromatoid bodies within each syncytium opens the question of whether developing germ cells have some control over the likelihood of a given molecule being shared or not.

## That sinking feeling: Can RNA trafficking explain how germ cells get to be choosy?

Prior to Umehara's report that TLR7/8 are restricted to X-bearing sperm, only three coding genes (Sperm motility kinase 2a/2b [*Smok2a/2b*], Sperm adhesion molecule [*Spam1*], and Sphingomyelin phosphodiesterase 1 [*Smpd1*]—all of them autosomal) have been reported to escape sharing in mice [20–22]. Conversely, only four genes have been explicitly shown to undergo sharing: X-linked A-kinase anchoring protein 4 (*Akap4*) [9], autosomal protamine 1 (*Prm1*) [23], and two reporter transgenes expressed under spermatid-specific promoters [8,24].

We posit that transcript sharing between syncytial germ cells can be considered under a source–sink model, beginning with transcription in the nucleus, and ending when the RNA is degraded. Thus, the degree of sharing of any transcript must depend on its life cycle—its level of transcription, nucleocytoplasmic export, diffusion and/or directed trafficking within the cytoplasm, and degradation. This model predicts different degrees of sharing for different classes of RNAs. In particular, slower diffusion within the cytoplasm and/or more-rapid RNA degradation must lead to a steeper gradient of RNA with higher concentrations near the source, i.e., more RNA being retained within the transcribing cell. Thus, RNAs that are either retained within the nucleus or rapidly degraded within the cytoplasm (e.g., most long noncoding RNA (lncRNA), small nuclear RNA (snRNA), and pseudogenes subject to nonsense-mediated decay) are expected to be more likely to escape sharing. Conversely, rapidly diffusing, cytoplasmic RNA species (such as mRNA, transfer RNA [tRNA], micro RNA [miRNA], and Piwi-interacting RNA [piRNA]) will spread more readily through the syncytium and thus appear more evenly shared. However, we note that some mRNAs, particularly those encoding organelle-targeted and membrane-associated proteins, show directed trafficking and spatially restricted translation in a range of cell types [25]. This may constrain their diffusion and reduce the level of transcript sharing between germ cells. Similar source–sink considerations apply to gene product sharing at the protein level, except that the site of production is the ribosome. In this case, because the selectivity will be exerted at the protein level, the mRNA can be equally distributed across the cyst. Transcripts might subsequently be selectively translated only in specific cells or subject to differential protein degradation, posttranslational modification, or protein trafficking. An underlying assumption of this process, however, is that some other gene product(s) must also be asymmetrically distributed within the cyst in order to enforce this type of regulation [26].

## You don't need to follow anybody… You're all individuals!

A recent preprint of a single-cell sequencing study has provided evidence for the sink–source model proposed above, suggesting that as many as 29% of all spermatid-expressed autosomal genes may show some degree of incomplete sharing [27]. Importantly, as predicted from the model, nuclear and NMD-targeted RNAs are more likely to escape sharing (Robin Friedman, personal communication), and the chromatoid body is enriched for fully shared mRNAs. Thus, it is possible that TLR7/8, rather than exceptions, are in fact part of a larger class of gene products that are unequally shared across the sperm cell syncytium.

**Fig 2:**
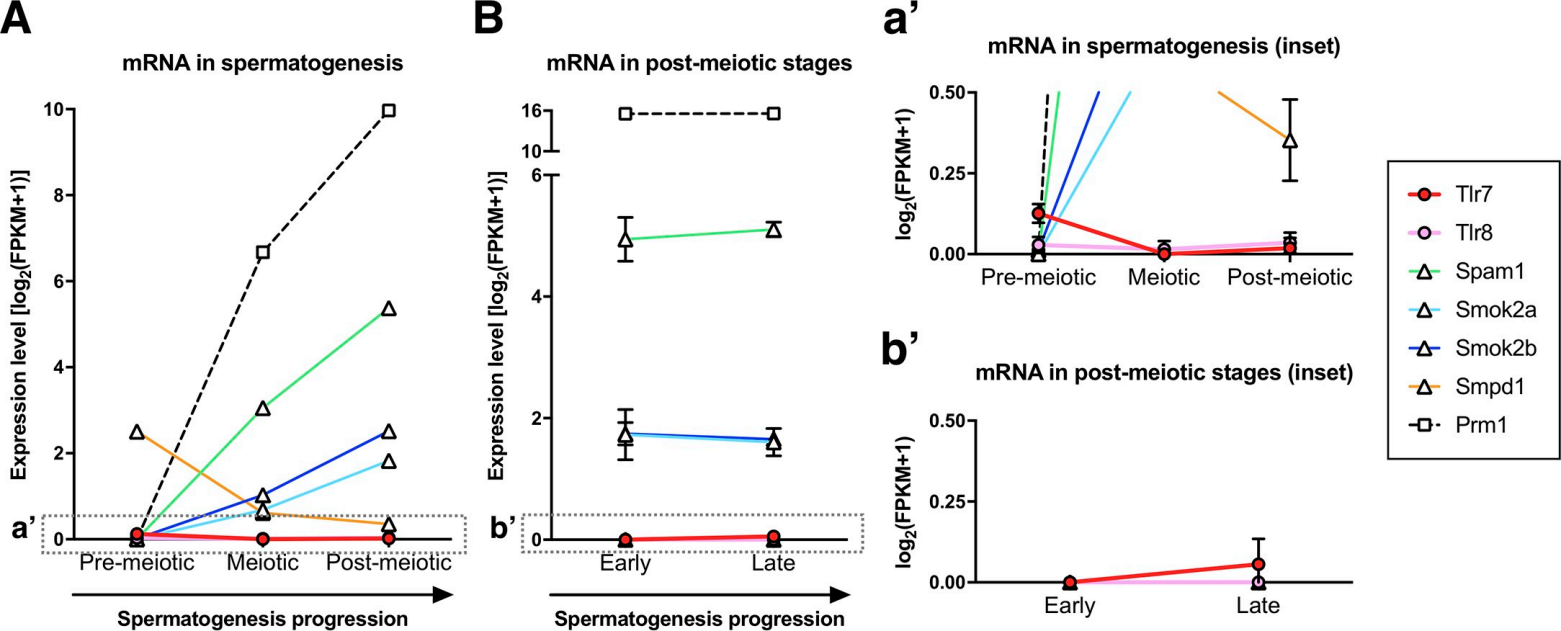
*Tlr7/8* transcripts are detected at extremely low levels during spermatogenesis. (A) mRNA levels in an RNAseq data set analysing different stages of pre- to postmeiotic sperm cell development [28], measured in FPKM. Premeiotic corresponds to purified spermatogonia, meiotic corresponds to spermatocytes, and postmeiotic corresponds to round spermatids. Transcript levels were recalculated from raw data and are plotted as mean log_2_(FPKM+1) ± SD. *Tlr7/8* are depicted by coloured circles; previously reported genes that escape full sharing (*Spam1*, *Smok2a/2b*, and *Smpd1*) are indicated by triangles; and squares denote a well-shared postmeiotic control gene (*Prm1*). (B) mRNA levels in an RNAseq data set analysing two different stages of postmeiotic sperm cell development [29]. Early corresponds to round spermatids, and late corresponds to elongating spermatids. Transcript levels were recalculated from raw data and plotted in FPKM as in panel A. Inserts (a’) and (b’) provide additional detail on the low expression levels of TLR7/8. FPKM, fragments per kilobase of transcript per million reads; mRNA, messenger RNA; *Prm1*, protamine 1; RNAseq, RNA sequencing; *Smok2a/2b*, Sperm motility kinase 2a/2b; *Smpd1*, Sphingomyelin phosphodiesterase 1; *Spam1*, Sperm adhesion molecule 1; *Tlr*, Toll-like receptor.

The phasing approach employed by Bhutani and colleagues to identify transcripts with incomplete sharing relies on the allelic balance of autosomal SNPs in individual postmeiotic cells. Consequently, this method could not be applied to sex-linked genes. To identify these, Bhutani and colleagues used a pseudotime analysis followed by clustering via pairwise correlation. This failed to highlight TLR7/8 as being retained in X-bearing spermatids. By reanalysing two independent mouse spermatogenesis RNA sequencing (RNAseq) datasets [28–29], we observe that *Tlr7/8* transcripts are detected at extremely low levels irrespective of the sperm cells’ developmental stage (**Fig 2**). These observations raise two possibilities: firstly, *Tlr7/8* transcripts may be low level and/or rapidly degraded; alternatively, *Tlr7/8* mRNA may be tightly bound to some other cellular component and thus poorly extracted from the germ cells. Both of these are compatible with the source–sink model of transcript compartmentalisation.

Intriguingly, Umehara and colleagues found higher TLR7/8 protein levels in the transcriptionally silent mature sperm cells compared with the still transcriptionally active round/elongating spermatids. This observation indicates that some proportion of *Tlr7/8* transcripts must be long-lived enough to be stored for later translation. This ‘storage’ could take the form of aggregate structures that restrict sharing (as in the case of *Smok* genes [20]), highlighting the functional relevance of future studies on RNA/protein interactions in postmeiotic cells. We note that because Umehara and colleagues did not report the localisation of *Tlr7/8* transcripts, we cannot formally exclude the possibility that the low levels of the mRNA are widely shared but that the protein is selectively translated/degraded in a cell type–specific manner. However, a further suite of X/Y cell–specific products would be required to enforce this (as argued above and in [26]).

## Do TLR7/8 take a toll on viral infections in the reproductive tracts?

The mechanism through which TLR7 and TLR8 might achieve differential accumulation in developing germ cells represents just one layer of the mystery behind their possible involvement in reproduction. What function could these immunity-related genes play in the context of the germ line, and what could be the evolutionary consequences of their apparently exclusive association with X-bearing male gametes?

Umehara and colleagues speculate that TLR7/8 could be activated in response to viral infection either of the male or female reproductive tract. Indeed, in somatic cells, TLR7/8 are a part of the innate immune system involved in recognising and combating viral infections. More specifically, they mediate via the binding of single-stranded RNA (ssRNA) the initiation of the immune response against this class of infectious agents [30–31]. As hypothesised by Umehara and colleagues, the function of TLR7/8 may be to slow down sperm that were exposed to viruses during their passage through the male or female reproductive tracts, thus protecting the future embryo from infection. Yet there are several nuances to this hypothesis that should be taken into consideration.

## Changing places: The localisation and function of TLR7/8 in male germ cells

One nuance relates to the possible functions of TLR7/8 in the germ line. In somatic cells, TLR7/8 are mainly endosomal (i.e., intracellular) receptors that require proteolytic cleavage within the endolysosome in order to bind their ligand (ssRNA) [32]. This ensures they are only activated by viral nucleic acids and not by endogenous cellular ssRNAs. The extent to which these rules could apply to male germ cells is not entirely clear. Mature sperm cells are assumed to be incapable of endocytosis [33] and thus are not expected to mount an endosomal response to viral RNA. In principle, if a cell surface version of TLR7/8 were to be present and active in mature male gametes, it could respond to free ssRNA (though not to intact virus particles). Regarding this point, the data of Umehara and colleagues on the localisation of TLR7/8 in mature sperm cells are inconclusive. Because their staining was performed on fixed, permeabilised sperm cells, it is impossible to know whether TLR7/8 are bound to internal cell membranes (as seen in somatic cells) or located on the cell surface. In this regard, we should also consider the possibility that the functions of TLR7/8 in the germ line, and not just their localisation, may deviate from those in somatic cells. Under such a scenario, germ line TLR7/8 (either at the cell surface or intracellularly) could be activated by some other nucleotide-related signalling molecule synthesised by the male or female reproductive tracts, leading to functional changes in the gametes. However, the most puzzling aspect of this story emerges when we consider the evolutionary consequences of a potentially exclusive functional association between TLR7/8 and X-bearing gametes.

## Something here doesn’t fit …

The existence of X-linked genes that selectively impair X-bearing sperm during fertilisation presents an evolutionary conundrum. Such genes would act as sex ratio distorters, reducing the fertilisation success of gametes carrying the X chromosome and yielding male-biased progeny. Outside of cytoplasmic sex ratio distortion (e.g., *Wolbachia* [34]), most described sex ratio distorters are X- or Y-linked selfish genetic elements that take advantage of the lack of sequence homology between sex chromosomes to increase their frequency in the population (at the expense of the alternate chromosome) [35]. Although the specific molecular mechanisms used by sex ratio distorters vary, they all impair the fitness of sperm bearing the homologous sex chromosome—either during meiosis by altering the ratio of gametes produced, postmeiosis by altering the sperm function, or postfertilisation by altering offspring viability [36]. However, both theory and data suggest that we should not expect to find X-linked genes that selectively inhibit the function of X-bearing rather than Y-bearing sperm, as such alleles are evolutionarily suicidal and should be rapidly eliminated from a population via natural selection [2].

Perhaps the simplest answer to this question is that the TLR7/8 receptors have no in vivo function in germ cells. For example, maybe TLR7/8 are not activated under physiological conditions, because of the absence of endogenous ligands in the female reproductive tract, or their activation has no functional significance on sperm motility in vivo. The plausibility of this solution is supported by the fact that TLR7/8 are not required for natural fertility (see below). Although this hypothesis resolves the evolutionary conundrum, it does raise further mechanistic questions about these genes. If TLR7/8 have no germ line function, why are they expressed during germ cell development, and why are the mRNAs or receptor proteins not shared between X- and Y-bearing germ cells?

## The evolutionary significance of TLR7/8-mediated sex ratio distortion: Cui bono?

Alternatively, if TLR7/8 do function in germ cells, this function would likely have a differential impact on X- and Y-bearing sperm (i.e., TLR7/8 would be engaged in genetic conflict). We consider below which genomic factions might benefit from such a putative conflict.

First, perhaps TLR7/8 are activated during natural reproduction, but this activation ultimately benefits X-bearing sperm. For example, if the functional consequences of activation are dose dependent, it is conceivable that low levels of receptor ligation rather than the high levels used in the IVF experiments somehow increase the motility of X-bearing sperm. Another possibility is that reduced sperm cell motility is in itself beneficial in some contexts. During IVF, sperm cells are processed to acquire the fast motility that defines the fertilisation-capable (capacitated) state. Yet in vivo, sperm cells may need to survive for several days in the female reproductive tract before fertilisation [37]. In these cases, ‘precocious’ capacitation can be detrimental to the success of sperm cells. In other words, fertilisation is sometimes a marathon rather than a sprint. In these scenarios, TLR7/8 function could be interpreted as that of classic X-linked selfish genes.

Second, one can hypothesise a scenario in which TLR7/8 receptor activity might be detrimental to all meiotic products, and Y-bearing cells are unresponsive to or protected from this ‘poison’. Such protection could result from a Y-linked ‘antidote’ gene that confines TLR7/8 mRNA or protein to X-bearing cells or that selectively degrades TLR7/8 message or protein in Y-bearing cells. In this scenario, the antidote itself could be freely shared between both X- and Y-bearing cells because under the source–sink model (see above), any factor that impairs free diffusion or promotes degradation of TLR7/8 transcripts will lead to an increased concentration of mRNAs at their site of production (X-bearing cells). Such an antidote would then constitute a Y-linked sex ratio distorter, with TLR7/8 as its X-linked ‘target’.

Third, the restriction of TLR7/8 to X-bearing sperm could allow mothers to modulate the sex ratio of their progeny via regulation of the receptor ligand in the female reproductive tract [38]. In this scenario, expression of the ligand could impair X-bearing sperm in conditions in which male-biased litters maximise a female's expected fitness and/or conceivably favour X-bearing sperm and female-biased litters, as hypothesised above.

## The young and the restless: What can evolutionary studies tell us?

Although sex ratio distorters increase the fitness of the sex chromosome on which they reside, the fertility costs associated with the functional impairment of half of the sperm cell population deposited in the female reproductive tract combined with the benefits of producing the rarer sex in a population with a biased sex ratio impose strong selective pressure to evolve suppressors. Thus, most known sex ratio distorters are ‘cryptic’ and suppressed by loci on the autosomes or the other sex chromosome [39].

**Fig 3:**
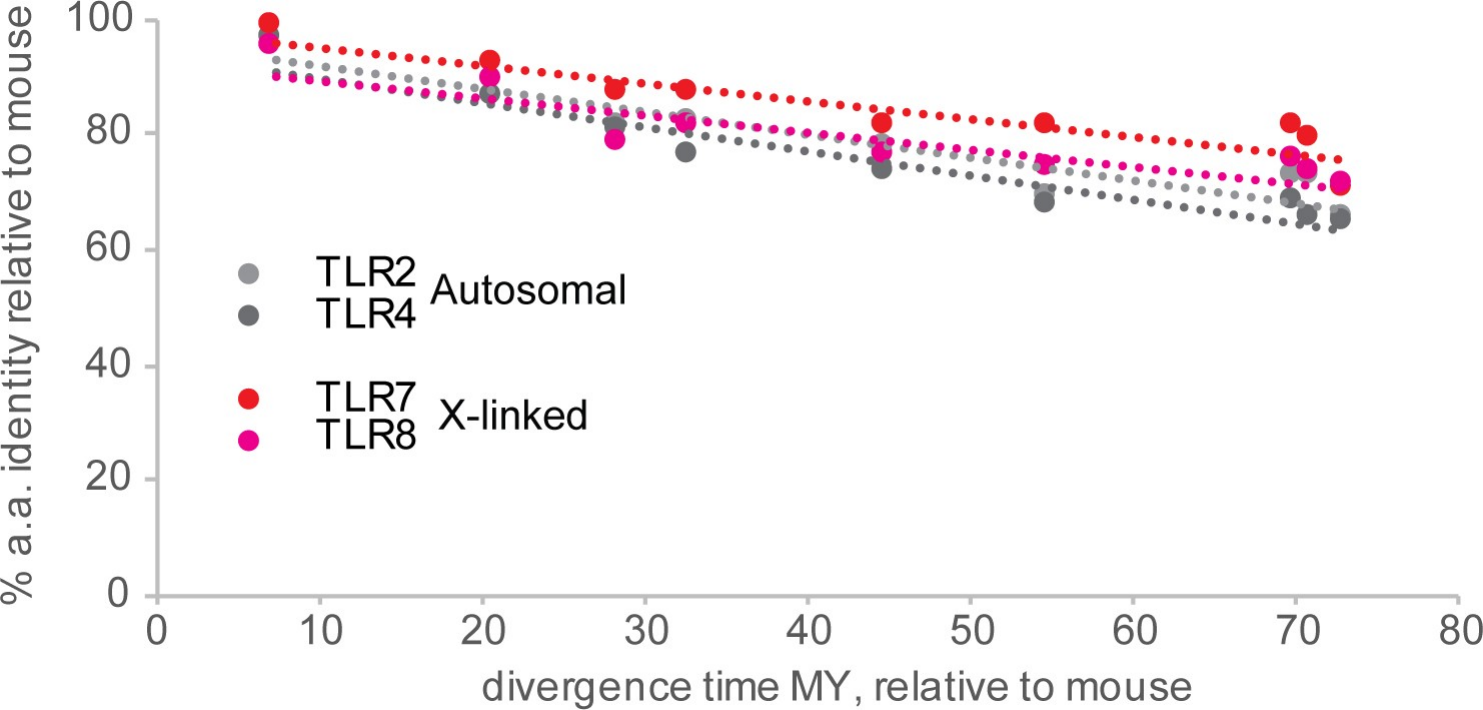
Percent amino acid identity between TLR orthologs of increasing evolutionary divergence. Autosomal TLR2, TLR4 (grey), and X-linked TLR7 and 8 (red and pink) are shown. Each data point corresponds to the percent pairwise identity between mouse (*Mus musculus*) and the following species: *Mus caroli* (7.4 MY), *Rattus norvegicus* (20.9 MY), *Meriones unguiculatus* (28.6 MY), *Microtus ochrogaster* or *Mesocricetus auratus* (33 MY), *Spalax galili* (45 MY), *Jaculus jaculus* (54.8 MY), *Castor canadensis* (70 MY), *Ictidomys tridecemlineatus* (71 MY), and *Cavia porcellus* (73 MY). MY, million years; TLR, Toll-like receptor.

As with other systems of genetic conflict, these complex antagonistic interactions have been shown to lead to rapid coevolution between drivers, targets, suppressors, and enhancers of drive [40]. If TLR7/8 are engaged in such a genetic conflict, we would therefore expect that features of this system would be evolutionarily young, dynamic, and/or unstable. More specifically, if TLR7/8 function as an X-linked sex ratio distorter, we should expect to find Y-linked or autosomal suppressors, as has been observed for most documented sex ratio systems [38–41]. Similarly, if TLR7/8 are targets of a Y-linked distorter or of maternal manipulation, we would expect to see rapid molecular evolution of TLR7/8 sequences to either counter the drive or inactivate the genes altogether. Although TLR8 has previously been reported as one of the most diversified TLRs [42], a focused comparison of evolutionary rates within rodents reveals comparable rates of evolution between the X-linked TLR7/8 and the autosomal TLR2 and TLR4 (**Fig 3**, with estimated median divergence times measured in million years [MY] according to timetree.org [43]). Although this observation fails to provide support for rapid molecular evolution of TLR7/8 relative to other TLRs, an important caveat is that the entire TLR gene family is rapidly evolving [44]. This is hypothesised to result from their immune functions that ultimately engage them in a genetic conflict with pathogens [45–46].

It is worth noting that TLR7/8 are X-linked in a wide range of mammals, including human, mouse, cattle, and cats, and thus have probably not moved out of the X in over 100 MY. This observation is potentially at odds with several of the above-outlined genomic conflict scenarios because these would tend to select for loss of TLR7/8 from the X. However, the retention of TLR7/8 in the genome could potentially be explained by antagonistic pleiotropy, and translocation to an autosome might also be disfavoured because these genes are sensitive to both over- and underdosage [31,47–49]. A useful avenue for future studies will be to test the phylogenetic limits of the sex-skewing protocol devised by Umehara and colleagues—which species are amenable to this, and how does this relate to TLR expression patterns in each species?

## Back to the lab: What can mechanistic studies tell us?

As outlined above, the simplest resolution of the mystery is that the in vivo response to TLR7/8 activation differs from the effects reported in the IVF experiments. This question can only be resolved by understanding the endogenous activation pattern of TLR7/8 during fertilisation. Umehara and colleagues utilised different doses and incubation times for their drug treatments and saw no evidence of a biphasic effect; however, other evidence has suggested that different TLR ligands can engage functionally different downstream pathways [50]. Thus, it remains possible that the physiological ligand for sperm-borne TLR7/8—whether RNA based or some other signalling molecule—acts to promote rather than impede sperm fertilising capacity. Inroads into this question might come from investigating models in which the dams are known (or predicted) to manipulate offspring sex ratio, such as in high versus low body condition. In particular, does oviductal fluid from dams reared under different conditions selectively affect X- and Y-bearing sperm motility?

Transgenic and/or mutant mouse models provide another avenue to validate the in vitro observations. In particular, deficiency for TLR7, TLR8, or both should abolish the effect of agonist treatment on sex-specific sperm motility. Knockout lines for both genes have already been characterised and are fertile, with no reports of skewed progeny sex ratios [31,47]. As a further comparator, the Y autoimmune accelerator (*Yaa*) mouse has an X > Y translocation placing an additional copy of TLR7 and TLR8 on the Y chromosome [48,49]. Thus, *Yaa* animals should show impairment of both X and Y sperm motility in response to treatment, whereas combining a *Yaa*-derived Y chromosome with deletion of the X-linked TLR7/8 copies should reverse the response to treatment.

A final experimental system that may be of use in studying this matter are male mice with deletions on the long arm of the mouse Y chromosome (Yqdel males). Yq deletions lead to skewed sex ratios, with the extent of the skew correlating with the extent of the deletion. There is a wide body of work on paternal control of mouse sex ratio, including not only the use of these deletions but also analysis of inter-subspecific hybrids and population genetics studies of X/Y amplicon dynamics. These studies collectively show that mouse sex ratio is regulated by a competition between the ampliconic sex-linked transcriptional regulators Sycp3-like X-linked (*Slx*), Slx-like 1 (*Slxl1*), and Sycp3-like Y-linked (*Sly*) [12]. Loss or knockdown of *Sly* leads to up-regulation of X-linked genes and female-biased litters as a result of differential X/Y sperm motility [51], but the causal chain between *Slx/Sly* competition and sperm motility remains elusive. Could TLR7/8 be the missing link? Because Yq-deleted males overexpress X-linked genes and overtransmit the X chromosome, this would suggest that if TLR7/8 is the effector mechanism in this conflict, then in vivo TLR7/8 activity favours X transmission (as in one of the evolutionary scenarios described above). Understanding how Yqdel sperm react to TLR7/8 agonists and/or comparing TLR7/8 transcript sharing patterns between wild-type and Yqdel males may yield further insight.

## Conclusion

We have presented several considerations regarding our mechanistic and evolutionary understanding of TLR7/8 activity and their potential role in sperm biology. Having addressed this problem from the intersection between developmental and evolutionary biology, we conclude that TLR7/8 should not reduce the fitness of X-bearing sperm in vivo. Thus, absent new revelations regarding the role of these genes in germ cell biology, we favour the view that any effects of TLR7/8 activity in vivo must differ from the in vitro findings of Umehara and colleagues. In light of recent data strongly suggesting that incomplete transcript sharing may be a wider phenomenon than previously anticipated, the most parsimonious explanation for the TLR7/8 conundrum is that both genes escape sharing because their high mRNA turnover limits diffusion across the sperm cell syncytium, and this asymmetric distribution is not selected against because both receptors have no functional significance for in vivo fertilisation. This may for example be the case if they turn out to be intracellular receptors that are only ever exposed to ligands following in vitro gamete manipulation. Despite these considerations, the work of Umehara and colleagues opens exciting avenues for further research into a considerably overlooked aspect of reproductive biology: the possibility of haploid selection in multicellular animals.

## Abbreviations

Akap4: A-kinase anchoring protein 4
FPKM: fragments per kilobase of transcript per million mapped reads
IVF: in vitro fertilisation
lncRNA: long noncoding RNA
mRNA: messenger RNA
miRNA: micro RNA
MY: million years
piRNA: Piwi-interacting RNA
Prm1: protamine 1
RNASeq: RNA sequencing
Slx: Sycp3-like X-linked
Slxl1: Slx-like 1
Sly: Scyp3-like Y-linked
Smok2a/2b: Sperm motility kinase 2a/2b
Smpd1: Sphingomyelin phosphodiesterase 1
snRNA: small nuclear RNA
Spam1: Sperm adhesion molecule 1
ssRNA: single-stranded RNA
TLR: Toll-like receptor
tRNA: transfer RNA
Yaa: Y autoimmune accelerator
Yqdel: deletions on mouse chromosome Yq.
